# Supplementary material for: Non-inferiority of deep learning ischemic stroke segmentation on non-contrast CT within 16-hours compared to expert neuroradiologists
Source: Sci Rep. 2023 Sep 26;13:16153. doi: 10.1038/s41598-023-42961-x (PMC10522706; doi:10.1038/s41598-023-42961-x)
Supplement: Supplementary file 1 — Supplementary Information. [file 41598_2023_42961_MOESM1_ESM.pdf]

Supplemental information

Table S.1. Model optimization trained on Expert A on Validation Sets

Metric Category	Metric [2]	Expert A to **3D Baseline Model**[1]		Expert A to **2D Model**[1]		Expert A to **3D Tuned Model**[1]		Expert A to **3D plus Mirrored Input**[1]		Expert A to **3D Mirrored, Tuned Model**[1]	
**Volume**	**VS**	0.71	± 0.05	0.64	± 0.05	0.75	± 0.04	0.72	± 0.04	**0.77**	**± 0.04**
	**AVD [ml]↓**	5.58	± 1.07	4.95	± 1.09	5.06	± 0.85	4.91	± 0.98	**4.90**	**± 0.94**
**Overlap**	**Dice**	0.40	± 0.09	0.35	± 0.08	0.43	± 0.06	0.38	± 0.07	**0.45**	**± 0.06**
	**Precision**	0.46	± 0.08	**0.57**	**± 0.1**	0.44	± 0.09	0.50	± 0.06	0.49	± 0.07
	**Recall**	0.36	± 0.08	0.30	± 0.07	0.41	± 0.08	0.34	± 0.09	**0.45**	**± 0.08**
**Distance**	**HD 95 [mm]↓**	17.12	± 3.82	19.64	± 4.44	16.44	± 2.34	16.88	± 4.34	**15.81**	**± 2.69**
	**SDT $_{5mm}$**	0.65	± 0.05	0.64	± 0.06	0.67	± 0.06	0.64	± 0.06	**0.67**	**± 0.05**

[1]Median ± 95% CI (bootstrapped). Bold values represent the highest and underlined values display a significant increase in performance compared to the 3D baseline model.
[2] VS = Volumetric Similarity, AVD = Absolute Volume Difference, HD 95 = Hausdorff Distance 95th percentile, SDT = Surface Dice at Tolerance

Table S.2. Comparison of Model trained on Expert C and compared to Experts B and A on Test Sets

Categories	Metric [1]	Expert B			Expert A			Expert C
		**Inter-Expert**[2] (B to C)	**Model-Expert**[2] (B to Model)	**p-value**[3] for non-inferiority	**Inter-Expert**[2] (A to C)	**Model-Expert**[2] (A to Model)	**p-value**[3] for non-inferiority	**Model-Expert**[2] (C to Model)
**Volume**	**VS**	0.36 ± 0.24	0.49 ± 0.16	p<0.01	0.58 ± 0.28	0.58 ± 0.07	p<0.01	0.71 ± 0.15
	**AVD [ml]**	8.36 ± 4.67	8.59 ± 5.34	non-sig	7.28 ± 4.96	8.74 ± 5.19	non-sig	2.39 ± 2.59
**Overlap**	**Dice**	0.24 ± 0.19	0.32 ± 0.2	p<0.0001	0.25 ± 0.14	0.41 ± 0.19	p<0.01	0.45 ± 0.12
	**Precision**	0.14 ± 0.14	0.20 ± 0.18	p<0.001	0.16 ± 0.14	0.28 ± 0.13	p<0.01	0.44 ± 0.14
	**Recall**	0.73 ± 0.14	0.79 ± 0.19	p<0.001	0.61 ± 0.17	0.70 ± 0.25	p<0.01	0.60 ± 0.23
**Distance**	**HD 95 [mm]**	18.63 ± 8.61	21.32 ± 8.08	non-sig	21.43 ± 7.35	27.19 ± 8.76	non-sig	17.81 ± 9.59
	**SDT 5mm**	0.41 ± 0.25	0.43 ± 0.17	p<0.01	0.40 ± 0.12	0.51 ± 0.18	p<0.01	0.53 ± 0.11

[1] VS = Volumetric Similarity, AVD = Absolute Volume Difference, HD 95 = Hausdorff Distance 95th percentile, SDT = Surface Dice at Tolerance
[2] Median ± 95% CI (bootstrapped)
[3] p-values of one-sided Wilcoxon sign rank test

Table S.3. Comparison of Model trained on Expert B and compared to Test Experts A and C on Test Sets

Categories	Metric [1]	Expert A			Expert C			Expert B
		**Inter-Expert**[2] (A to B)	**Model-Expert**[2] (A to Model)	**p-value**[3] for non-inferiority	**Inter-Expert**[2] (C to B)	**Model-Expert**[2] (C to Model)	**p-value**[3] for non-inferiority	**Model-Expert**[2] (B to Model)
**Volume**	**VS**	0.65 ± 0.1	0.78 ± 0.14	p<0.0001	0.36 ± 0.25	0.52 ± 0.3	p<0.0001	0.80 ± 0.07
	**AVD [ml]**	7.62 ± 4.4	4.68 ± 4.67	non-sig	8.36 ± 4.82	5.02 ± 2.88	p<0.05	5.43 ± 3.81
**Overlap**	**Dice**	0.47 ± 0.17	0.41 ± 0.15	p<0.01	0.24 ± 0.19	0.25 ± 0.12	p<0.0001	0.55 ± 0.15
	**Precision**	0.60 ± 0.18	0.46 ± 0.15	non-sig	0.73 ± 0.14	0.63 ± 0.22	p<0.01	0.48 ± 0.17
	**Recall**	0.47 ± 0.28	0.40 ± 0.17	p<0.001	0.14 ± 0.14	0.15 ± 0.14	p<0.0001	0.67 ± 0.15
**Distance**	**HD 95 [mm]**	15.51 ± 5.21	21.49 ± 6.35	non-sig	18.63 ± 8.23	19.69 ± 7.15	non-sig	11.63 ± 3.85
	**SDT 5mm**	0.48 ± 0.22	0.44 ± 0.16	p<0.05	0.29 ± 0.18	0.25 ± 0.13	p<0.0001	0.68 ± 0.1

[1] VS = Volumetric Similarity, AVD = Absolute Volume Difference, HD 95 = Hausdorff Distance 95th percentile, SDT = Surface Dice at Tolerance
[2] Median ± 95% CI (bootstrapped)
[3] p-values of one-sided Wilcoxon sign rank test

Table S.4. **Modification of nnUNet**

Category	Configuration	Details	Conclusion
**Filters**	2D vs. 3D	NCCT scans of the head are commonly anisotropic causing the sagittal and coronal view to be low resolution.	The 2D model required less training time (7 hours per fold) but had a lower SDT_{5mm} than the 3D model. These results are contrary to previous works, that proposed 2D over 3D filter configurations[1,2], because of lower memory consumption, model complexity, and skewed receptive field for segmentation of anisotropic images.
**Input**	Mirrored co-registered input image	Several studies suggest a benefit of symmetry awareness for the assessment of NCCTs of the head with CNNs[1,3,4]. Together with the original image we input the co-registered image, which elevates computational cost while providing contra-lateral information.	A model that included a mirrored co-registered image as a second channel to provide the network with symmetry information of the contralateral hemisphere also resulted in an improvement compared to the baseline model (SDT_{5mm} 0.66±0.05)
**Loss function**	Dice + Focal Entropy	The small lesion size (median of 12, IQR 5 - 30 ml) represents an inherent high-class imbalance within the image. For this reason, we used Dice + Focal Entropy with equal weights and alpha of 0.5 and gamma of 2. This increases the weight of positive voxels in the reference mask.[5]	
**Regularization techniques**		We further improved performance with increased L2 regularization, dropout of 0.1, a momentum of 0.85, and by setting the number of epochs to 350.	More regularization decreases the chance of overfitting to the variability of experts' ground truth segmentation.

Table S.5. **Definitions of Performance Metrics for Medical Image Segmentation**

Category	Metric	Abbreviation	Definition
**Volume**	Volumetric Similarity	VS	$1 - \frac{\left\| \|\hat{V}\| - \|V\| \right\|}{\|\hat{V}\| + \|V\| + \varepsilon}$
	Absolute Volume Difference	AVD	$\frac{1}{m}\sum_{i=1}^{m} \left\| V^i - \hat{V}^i \right\|$
**Overlap**	Dice Similarity Coefficient	Dice	$\frac{2\times TP}{2\times TP+FN+FP}$
	Recall = Sensitivity	Recall	$\frac{TP}{TP+FN}$
	Precision	Precision	$\frac{TP}{TP+FP}$
**Distance**	Hausdorff Distance, q = 95th percentile	HD 95	$\max\left(h(A,B), h(B,A)\right)$ with $h(A,B) = \max_{a\in A}\min_{b\in B} \|\|b-a\|\|$
	Surface Dice at Tolerance	SDT	$\frac{\|\hat{S}\cap B^t\| + \|S\cap \hat{B}^t\|}{\|\hat{S}\| + \|S\|}$

V=ground truth volume, $\hat{V}$ = predicted volume, TP= True Positive voxels, TN= True Negative voxels, FP= False Positive voxels, FN= False Negative voxels, S=surface voxels of ground truth, $\hat{S}$= predicted surface voxels, B^t= border volume of ground truth defined by tolerance t, $\hat{B}^t$= predicted border volume defined by tolerance t

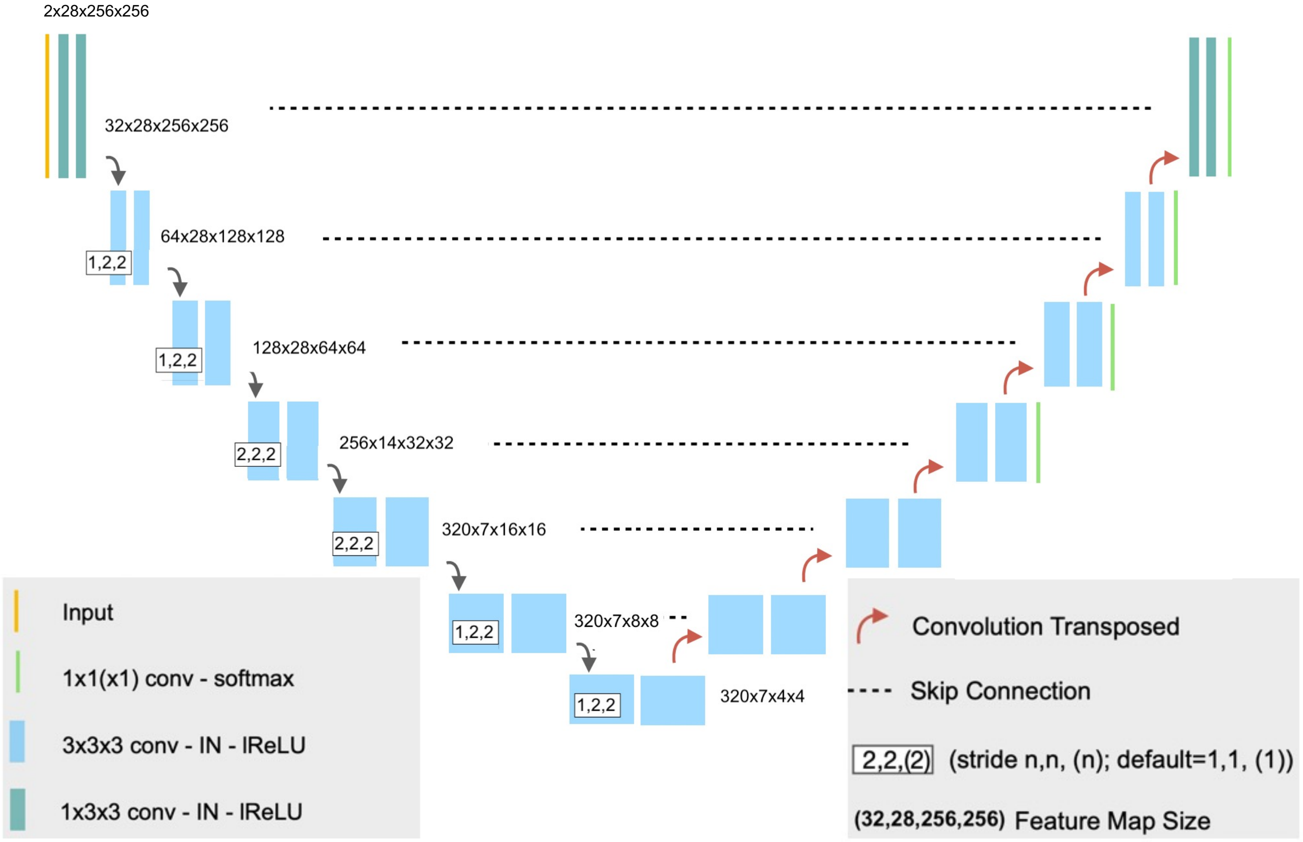

Figure S.1. **Model Architecture**

The modified nnUNet configuration includes a large patch size of 28x256x256, 7 stages with two 3D convolutions per stage. The preprocessed input had 2 channels: the CT image and a mirrored CT image in which the ipsilateral hemisphere is replaced by a mirrored version of the contralateral hemisphere. The output of the model was a segmentation mask), a spacing of (3.00, 0.45, 0.45) and dimensions of 22-56 x 512 x 512. The parameter space after each stage is denoted in pytorch's tensor convention (channels x depth x height x width)

.

Non contrast CT ischemic stroke outlining instructions

What you will be doing as a rater:

NCCT segmentation: Outline the NCCT lesion as precisely as you can. However, do not waste your time overthinking it. Since the hypodensities are often subtle, it is hard to draw these outlines. We do not want you to get stuck redrawing or trying to correct outlines when there is no clear right way to draw the outline. So, do the best you can, and then move on to the next case.

The outlining will be done in the Horos DICOM viewer which is Mac only. You can use the Mac at the Stroke Center if you don't have access to one.

A few general and important points:

- All strokes are M1, or ICA occlusions and you should keep this in mind when you read the cases.
- There will be cases where you don't see a lesion. In such cases please just draw a circular outline outside the brain. Then we know you reviewed the case and not accidentally skipped it.
- The outlines will be handled anonymously for the purpose of all analyses and reports. You will be known as rater A, B or C.

Horos setup:

1) Install Horos download from my box. Use version Horos 4.0.0.
2) Download the NCCT folder from Box also. Put it where you like.
3) Open Horos and create a blank database.

NCCT rating:

1) Start with the NCCT outlining by selecting File->Open Database folder from Horos, point to the NCCT folder and click Open. See **1_ReadInNCCTData.mov.**
    a) Now go through the cases sequentially and outline using the pen tool (shortcut D). See the **2_NCCToutlining.mov**
    b) Only outline regions that you believe are consistent with an ischemic stroke within 16h.
    c) In some cases, there is likely no discernible infarct. Draw a small circle outside the brain to indicate that you have read the case but found no lesion.
    d) When you are done, simply go to the next case. Your ROI is automatically stored.
    e) Add comments if there is something about this case you would like to convey. It could be anything. Use the comment2 column for that See **3_comments.mov.**
    f) When all cases are done you export them as indicated below under Export

Figure S.2. Segmentation Instruction Document for Experts

References

1. Christensen, S. *et al.* Optimizing Deep Learning Algorithms for Segmentation of Acute Infarcts on Non-Contrast Material-enhanced CT Scans of the Brain Using Simulated Lesions. *Radiol Artif Intell* **3**, DOI: 10.1148/ryai.2021200127 (2021).

2. Qiu, W. *et al.* Machine Learning for Detecting Early Infarction in Acute Stroke with Non-Contrast-enhanced CT. *Radiology* **294**, 638–644 (2020).

3. Ni, H. *et al.* Asymmetry disentanglement network for interpretable acute ischemic stroke infarct segmentation in non-contrast ct scans. In *International Conference on Medical Image Computing and Computer-Assisted Intervention*, 416–426 (Springer, 2022).

4. Zhang, H., Zhu, X. & Willke, T. L. Segmenting brain tumors with symmetry. *arXiv preprint arXiv:1711.06636* (2017).

5. Yeung, M., Sala, E., Schönlieb, C.-B. & Rundo, L. Unified focal loss: Generalising dice and cross entropy-based losses to handle class imbalanced medical image segmentation. *Comput. Med. Imaging Graph.* **95**, 102026 (2022).
